# Myocardial ^123^I-*m*IBG scintigraphy in relation to markers of inflammation and long-term clinical outcome in patients with stable chronic heart failure

**DOI:** 10.1007/s12350-016-0697-7

**Published:** 2016-11-17

**Authors:** Derk O. Verschure, René Lutter, Berthe L. F. van Eck-Smit, G. Aernout Somsen, Hein J. Verberne

**Affiliations:** 1 Department of Nuclear Medicine, Academic Medical Center, University of Amsterdam, P.O. Box 22700, 1100 DE Amsterdam, The Netherlands; 2 Department of Cardiology, Zaans Medical Center, Zaandam, The Netherlands; 3 Departments of Respiratory Medicine and Experimental Immunology, Academic Medical Center, University of Amsterdam, Amsterdam, The Netherlands; 4 Cardiology Centers of the Netherlands, Amsterdam, The Netherlands

**Keywords:** Heart failure, cardiac sympathetic activity, planar ^123^I-*m*IBG myocardial scintigraphy, inflammation, prognosis

## Abstract

**Aim:**

Chronic heart failure (CHF) results in both increased cardiac sympathetic activity and myocardial inflammation. The aim of this study was to identify the relationship between severity of heart failure (i.e., NT-proBNP and LVEF), cardiac sympathetic activity (^123^I-*m*IBG scintigraphy), and measures of inflammation in subjects with stable, optimally treated CHF. In addition, the predictive value for cardiac events (i.e., ventricular arrhythmia, progression of CHF and cardiac death) of ^123^I-*m*IBG parameters and these inflammatory markers was evaluated.

**Materials and Methods:**

Fifty-five CHF patients (age 66.3 ± 8.0 years, 78% male, LVEF 22.4 ± 6.3) referred for cardiac ^123^I-*m*IBG imaging were included. At 15 minutes (early) and 4 hours (late) after i.v. administration of ^123^I-*m*IBG (185 MBq), planar images were acquired. Early Heart/Mediastinum (H/M) ratio, late H/M ratio, and ^123^I-*m*IBG washout (WO) were calculated. NT-proBNP and markers of inflammation (i.e., C-reactive protein (CRP), IL-1β, IL-6, IL-8, IL-10, IL-12p40, tumor necrosis factor-α (TNF-α), soluble (s)E-selectin, myeloperoxidase (MPO), plasminogen activator inhibitor-1 (PAI-1), tPA, tumor necrosis factor receptor (TNFR) 1 and 2, and interferon (IFN) α and β) were measured in blood plasma samples, taken just before ^123^I-*m*IBG administration.

**Results:**

Mean early H/M ratio was 2.12 ± 0.39, late H/M ratio was 1.84 ± 0.40, and ^123^I-*m*IBG WO was 13.0 ± 10.9. LVEF was the only independent predictor of late H/M ratio (adjusted *R* ^2^ = 0.100, *p =* 0.011). NT-proBNP was an independent predictor of ^123^I-*m*IBG WO (adjusted *R* ^2^ = 0.090, *p =* 0.015). CRP, IL12p40, TNF-α, sE-selectin, MPO, PAI-1, tPA, and TNFR2 were not related to late H/M ratio and ^123^I-*m*IBG WO. During a median follow-up of 34 months (2–58 months), 13 patients experienced a cardiac event [ventricular arrhythmia (4), progression of CHF (4), and cardiac death (5)]. Univariate Cox regression analysis showed that the risk of a cardiac event was associated with CRP (HR 1.047 [1.013–1.081]), NT-proBNP (HR 1.141 [1.011–1.288]), MPO (HR 0.998 [0.996–1.000]), and late H/M ratio (HR 0.182 [0.035–0.946]). Multivariate Cox regression analysis showed that only CRP, NT-proBNP, MPO, and IL-12p40 were predictors of a cardiac event.

**Conclusion:**

Inflammation and cardiac sympathetic activity seem not to be related in stable CHF patients. This is corroborated by the finding that they both provide prognostic information in this specific CHF population. The current findings should be regarded as insightful but preliminary.

**Electronic supplementary material:**

The online version of this article (doi:10.1007/s12350-016-0697-7) contains supplementary material, which is available to authorized users.

## Introduction

Chronic heart failure (CHF) is a complex syndrome characterized by increased activity of the sympathetic nervous system,^1^,^2^ increased NT-proBNP levels, and increased pro-inflammatory cytokines in plasma and myocardial tissue.^3^,^4^ Inflammation plays an important role in the pathogenesis and progression of many forms of heart failure (HF). With progression of CHF, the plasma levels of pro-inflammatory cytokines increase. In addition, cardiac sympathetic hyperactivity is associated with progression of CHF.^5^ However, there is only limited information on the relation between the sympathetic nervous activity and the inflammatory status in CHF patients.

Cardiac sympathetic activity can non-invasively be assessed with cardiac ^123^I-*meta*-iodobenzylguanidine (^123^I-*m*IBG). *m*IBG is a norepinephrine (NE) analog that shares the same presynaptic uptake, storage, and release mechanisms as NE. Radiolabeling of *m*IBG with ^123^I allows imaging with gamma cameras.^6^ The heart-to-mediastinum (H/M) ratio reflects presynaptic myocardial uptake of ^123^I-*m*IBG. The early H/M ratio reflects predominantly the integrity of sympathetic nerve terminals (i.e., number of functioning nerve terminals and intact uptake-1 mechanism). The late H/M ratio offers predominantly information about neuronal function resulting from uptake, storage, and release. The myocardial ^123^I-*m*IBG washout (WO) reflects predominantly neuronal integrity of sympathetic tone/adrenergic drive.^7^ The late H/M ratio and ^123^I-*m*IBG WO have been shown to be of clinical value, especially for the assessment of prognosis, in many cardiac diseases.^8^^–^ 12 Decreased late H/M ratio and increased ^123^I-*m*IBG WO are associated with poor outcome in subjects with CHF.^5^,^11^

The aim of this study was to identify the relationship between severity of HF (i.e., NT-proBNP and LVEF), cardiac sympathetic activity assessed with ^123^I-*m*IBG scintigraphy, and measures of inflammation in patients with stable CHF. In addition, we evaluated the prognostic value of these inflammatory markers and myocardial ^123^I-*m*IBG-derived parameters (i.e., late H/M ratio and ^123^I-*m*IBG WO).

## Material and Methods

### Subjects

Patients with stable CHF (New York Heart Association (NYHA) class II to III), who were referred for ^123^I-*m*IBG scintigraphy in their work-up for primary ICD implantation between 2010 and 2015 to the department of Nuclear Medicine of the Academic Medical Center in Amsterdam, were enrolled. The inclusion criteria were as follows: stable heart failure (i.e., no myocardial infarction, hospitalization, or progression of heart failure) at least 3 months before inclusion and treatment with optimal medical therapy according to the European HF guidelines including beta-blockers and angiotensin-converting-enzyme inhibitors (ACE-I) or angiotensin receptor blockers (ARB) and if necessary loop diuretics.^13^ Exclusion for participation was pregnancy or intolerance for iodine. All subjects provided written informed consent. The study was approved by the local institutional review board and conducted according to the principles of the International Conference on Harmonization–Good Clinical Practice.

### ^123^I-*m*IBG Scintigraphy Acquisition and Analysis

All patients continued their HF medication prior to ^123^I-*m*IBG scintigraphy. To block uptake of free ^123^I by the thyroid gland, subjects were pretreated with 250 mg oral potassium iodide 30 minutes before intravenous (IV) injection of 185 MBq ^123^I-*m*IBG (GE Healthcare, Eindhoven, the Netherlands). Fifteen minutes (early acquisition) and 4 hours (late acquisition) after administration of ^123^I-*m*IBG, 10-minutes planar images were acquired with the subjects in supine position using a gamma camera equipped with a medium-energy (ME) collimator.

**Figure 1:**
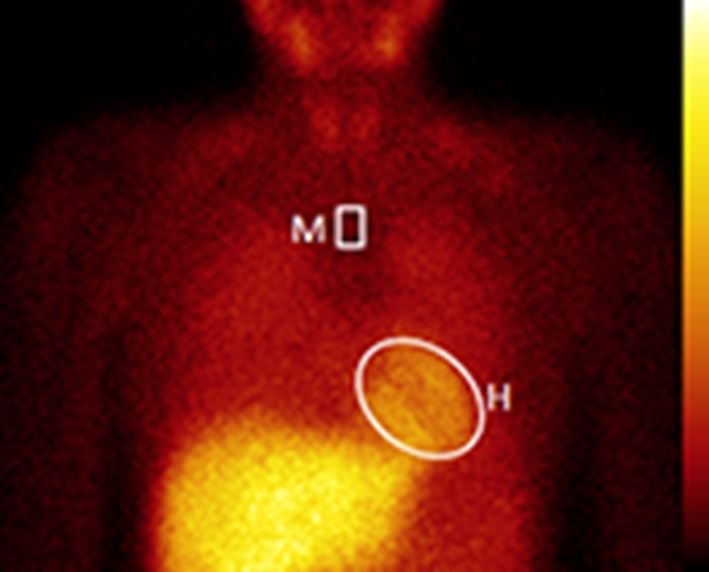
Example of processing procedure for planar ^123^I-*m*IBG images. The positioning of the mediastinal ROI was standardized in relation to the lung apex, the lower boundary of the upper mediastinum, and the midline between the lungs

All planar ^123^I-*m*IBG images were analyzed by one experienced observer (D.O.V.) blinded to patient data. Heart-to-mediastinum (H/M) ratios were calculated from the ^123^I-*m*IBG images using a region-of-interest (ROI) over the heart and the upper part of the mediastinum (Figure 1).^14^ The H/M ratio was calculated by dividing the mean count density in the cardiac ROI by the mean count density in the mediastinal ROI.^14^ The ^123^I-*m*IBG WO was calculated as follows:

$$ {\text{ WO }} = \left\{ {\frac{{ ( {\text{early H/M ratio) }} - ({\text{late H/M ratio)}}}}{\text{early H/M ratio}}} \right\}\,\; \times \; 100$$

### Markers of Inflammation

Before administration of 185 MBq ^123^I-*m*IBG, 2 × 4.0 mL of venous blood was collected into ethylenediaminetetraacetic acid (EDTA)-containing tubes. Immediately after collection of the required blood volume, one EDTA tube was analyzed for CRP and NT-proBNP by standard procedures. The second EDTA tube was mixed with the sampled blood and immediately cooled on ice. Within 30 min of collection, the tubes were centrifuged at 1700 g for 10 minutes. The plasma was transferred to cryovals and stored in upright position at -70 °C till analysis. All samples were analyzed at the end of the study for IL-1β, IL-6, IL-8, IL-10, IL-12p40, TNF-α, sE-selectin, MPO, PAI-1, tPA, TNFR1 and TNFR2, and INF α and β by luminex. Samples were measured with ProCarta reagents (eBioscience) following the supplier’s protocol and read on a Bioplex 200 (BioRad).

### Clinical Follow-up and Event Adjudication.

Follow-up was based on telephone interviews (D.O.V.) and medical records. All subjects received standard clinical care and were followed up until (1) the death of a subject was confirmed or (2) the trial was terminated. The Clinical Adjudication Committee reviewed data from case report forms and source documents to confirm the occurrence of CEs, specifically (1) HF progression: increase in symptomatic status from NYHA functional class II to III or IV, or increase from NYHA class III to class IV; (2) potentially life-threatening arrhythmic event, including documented episode of spontaneous sustained (30 seconds) ventricular tachyarrhythmia, resuscitated cardiac arrest, or appropriate ICD discharge (antitachycardia pacing or defibrillation); or (3) cardiac death (further classified as due to HF progression, sudden cardiac death [SCD]).

### Statistical Analysis

All continuous variables are expressed as mean ± standard deviation. After demonstrating a normal distribution of variables, between-group comparisons were performed using independent-sample *t* tests. Non-detectable levels of biomarkers were treated as zeroes for the analysis. Continuous data were compared using analysis of variance (ANOVA). The association between ^123^I-*m*IBG outcomes and biomarkers was assessed using Pearson’s correlation coefficient (2-tailed). Multivariate linear regression analysis was performed to determine independent predictors of ^123^I-*m*IBG parameters. The overall goodness-of-fit for each model was expressed as the adjusted R^2^. The F-test was used to assess whether a model explained a significant proportion of the variability. A *p* value < 0.05 was considered to indicate a statistical significance. Univariate and multivariate Cox proportional hazards regression analysis was used to assess independent predictors of cardiac events. ROC analysis was used to determine the optimal cut-off value (i.e., highest product of sensitivity and specificity) for predictors of CEs. Statistical analyses were performed with SPSS, release 22.0 for Windows (SPSS Inc., Chicago, IL, USA 2003).

## Results

In total, 252 patients with CHF were screened for enrollment. However, the majority of the subjects did not met the inclusion criteria (at least 3 months stable heart failure), already participate in other studies, or refused to give informed consent. Baseline patient characteristics including ^123^I-*m*IBG-derived parameters are presented in Table 1. The study population comprised 55 stable CHF patients (43 male and 12 female) with a mean age of 66.3 ± 8.0 years. Forty-one (75%) patients were in NYHA class II and 14 (25%) we in NYHA class III. The mean LVEF was 22.4 ± 6.3% and 60% of the patients had ischemic heart disease. The mean early H/M ratio was 2.12 ± 0.39, the late H/M ratio was 1.84 ± 0.40, and the mean ^123^I-*m*IBG WO was 13.0 ± 10.9. Most of the plasma levels of IL-1β, IL-6, IL-8, IL-10, TNFR1, IFN-α, and IFN-β were below the detection limit. Table 2 shows only biomarkers with levels above lower limit of detection in all patients.

**Table 1:** Baseline clinical characteristics of CHF patients

	All (*n* = 55)	Cardiac event (*n* = 13)	No cardiac event (*n* = 42)	*p* value
Age (years)	66.3 ± 8.0	66.4±7.0	66.2±8.4	0.118
Male (%)	43 (78)	10 (77)	33 (79)	0.809
Etiology heart failure				0.79
Ischemic (%)	33 (60)	8 (62)	25 (60)	
Non-ischemic (%)	22 (40)	5 (38)	17 (40)	
Blood pressure				
Systolic (mmHg)	125 ± 17	126 ± 14	125 ± 19	0.223
Diastolic (mmHg)	76 ± 10	77±8	75±11	0.148
LVEF (%)	22.4 ± 6.3	23.9 ± 6.6	22.1 ± 6.2	0.491
NYHA				0.051
Class II (%)	41 (75)	8 (62)	33 (79)	
Class III (%)	14 (25)	5 (38)	9 (21)	
Medication				
Betablockers (%)	43 (78)	9 (69)	34 (81)	0.118
ACE-I/ARB s(%)	50 (91)	13 (100)	37 (88)	0.004
MRAs (%)	18 (33)	5 (38)	13 (31)	0.560
Loop diuretics (%)	38 (69)	10 (77)	28 (67)	0.122
Statines (%)	38 (69)	9 (69)	29 (69)	0.980
Aspirin (%)	32 (58)	6 (46)	26 (62)	0.448
^123^I-*m*IBG parameters				
Early H/M ratio	2.12 ± 0.39	2.00 ± 0.28	2.16 ± 0.42	0.140
Late H/M ratio	1.84 ± 0.40	1.66 ± 0.28	1.90 ± 0.42	0.137
^123^I-*m*IBG WO	13.0 ± 10.9	19.8 ± 10.6	11.8 ± 10.8	0.897

**Table 2:** Biomarkers of CHF patients

	All (*n* = 55)	Cardiac event (*n* = 13)	No cardiac event (*n* = 42)	*p* value
CRP (mg/L)	5.44 ± 11.07	9.4 ± 20.3	4.2 ± 5.9	0.001
TNF-α (pg/mL)	6.00 ± 2.41	5.56 ± 1.41	6.12 ± 2.65	0.483
sE-selectin (ng/mL)	26.33 ± 10.67	28.91 ± 10.83	25.54 ± 10.62	0.800
IL-12p40 (pg/mL)	7.51 ± 5.58	9.68 ± 6.81	6.83 ± 5.04	0.154
MPO (ng/mL)	72.60 ± 56.44	52.05 ± 32.31	78.96 ± 60.95	0.018
PAI-1 (ng/mL)	8.50 ± 1.69	8.50 ± 2.04	8.51 ± 1.60	0.355
tPA (ng/mL)	1.56 ± 0.68	1.73 ± 0.83	1.51 ± 0.64	0.294
TNFR2 (pg/mL)	174 ± 57	191 ± 50	168 ± 58	0.602
NT-proBNP (ng/L)	1974 ± 3026	3280 ± 5402	1570 ± 1675	0.000

### Association Between ^123^I-*m*IBG and Markers of Inflammation

The LVEF, NYHA functional class, NT-proBNP, CRP, TNFα, TNFR2, sE-selectin, IL12p40, MPO, PAI-1, and tPA were used as explanatory variables of late H/M ratio and ^123^I-*m*IBG WO. Late H/M ratio was significantly associated with LVEF (*R* = 0.342, *p* = 0.011) and NT-proBNP (*R* = −0.272, *p* = 0.045). ^123^I-*m*IBG WO was also significantly associated with LVEF (*R* = −0.286, *p* = 0.034), NYHA (*R* = 0.281, *p* = 0.038), and NT-proBNP (*R* = 0.325, *p* = 0.015). Multivariate regression analysis using both biomarkers and clinical parameters (i.e., LVEF, NYHA functional class) showed LVEF as the only independent predictor of late H/M ratio (adjusted *R* ^2^ = 0.100, *p* = 0.011). NT-proBNP was the only independent parameter associated with ^123^I-*m*IBG WO (adjusted *R* ^2^ = 0.090, *p* = 0.015) (Table 3).

**Table 3:** Multivariate regression analysis to determine independent predictors for late H/M ratio (upper panel) and ^123^I-*m*IBG WO (lower panel)

Variabels	Coefficient *b*	Standard error *b*	*p* value
Constant	1.351	0.193	
LVEF	0.022	0.008	0.011
Goodness-to-fit of the model	Adjusted *R* ^2^		*p* value
	0.100		0.011

Variabels	Coefficient *b*	Standard error *b*	*p* value
Constant	10.667	1.678	
NT-proBNP	0.001	0.000	0.015
Goodness-to-fit of the model	Adjusted *R* ^2^		*p* value
	0.090		0.015

### Predictors of cardiac events

**Figure 2:**
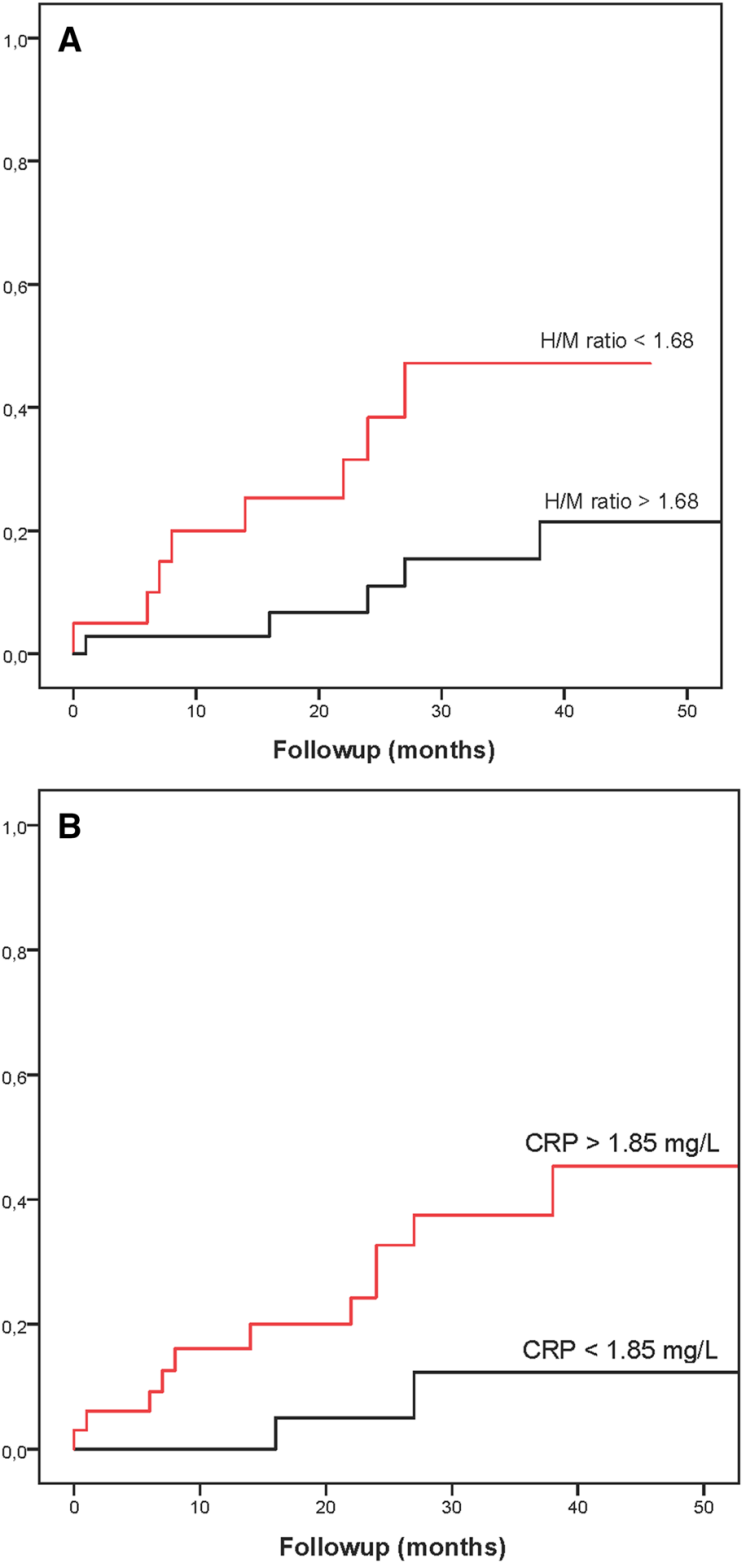
Examples of cumulative event curves for different parameters. **A** Comparing CHF patients with late H/M ratio <1.68 versus >1.68 (*p* = 0.019). **B** Comparing CHF patients with CRP < 1.85 mg/L versus CRP > 1.85 mg/L (*p* = 0.032)

None of the patients were lost during a median follow-up of 34 months (2–58 months). Thirteen patients (24%) experienced a first CE: progression of HF (*n* = 4), arrhythmic event with appropriate ICD discharge (*n* = 4), and cardiac death (*n* = 5; four subjects due to sudden cardiac death (SCD) and one due to progression of HF). In addition, one patient had a non-cardiac death. There was a significant difference in plasma levels of NT-proBNP, CRP, and MPO between patients with and without CEs (Table 2). However, there was no significant difference in late H/M ratio and ^123^I-*m*IBG WO between both groups. Univariate Cox regression analysis showed that the risk of a CE was associated with CRP, NT-proBNP, MPO, and late H/M ratio (Table 4). Multivariate Cox regression analysis showed that CRP, MPO, IL-12p40, and NT-proBNP were independent predictors of a CE. CRP, MPO, IL12p40, and NT-proBNP did not show any mutual relationship with each other (data not shown). Figure 2 shows cumulative event curves for the late H/M ratio and CRP. ROC analysis showed that the optimal cut-off values for the late H/M ratio and CRP were 1.68 and 1.85 mg/L, respectively. These cut-off values resulted in a moderate discriminative power: AUC for late H/M ratio 0.69 and for CRP 0.64, respectively).

**Table 4:** Univariate and multivariate Cox regression analysis for cardiac events

Variable	Univariate		Multivariate		
	HR (95% CI)	*p* value	HR (95% CI)	*X* ^2^ change	*p* value
CRP (mg/L)	1.047 (1.013–1.081)	0.000	1.050 (1.012–1.089)	4.767	0.009
NT-proBNP (ng/L)	1.141 (1.011–1.288)	0.023	1.177 (1.013–1.367)	3.330	0.034
IL-12p40 (pg/mL)	1.072 (0.980–1.173)	0.123	1.158 (1.045–1.283)	5.942	0.005
MPO (ng/mL)	0.985 (0.971–0.999)	0.030	0.981 (0.965–0.995)	5.253	0.014
LVEF (%)	1.033 (0.949–1.123)	0.452			
NYHA	2.096 (0.683–6.426)	0.186			
TNF-α (pg/mL)	0.787 (0.508–1.219)	0.302			
sE-selectin (ng/mL)	1.009 (0.958–1.063)	0.730			
PAI-1 (ng/mL)	0.892 (0.626–1.271)	0.528			
tPA (ng/mL)	1.059 (0.468–2.396)	0.890			
TNFR2 (pg/mL)	1.002 (0.992–1.011)	0.743			
Early H/M ratio	0.323 (0.065–1.600)	0.166			
Late H/M ratio	0.182 (0.035–0.946)	0.042			
^123^I-*m*IBG WO	1.045 (0.987–1.106)	0.136			

## Discussion

The findings of this study show that in this specific, optimally treated and stable CHF population, general markers of inflammation were not associated with increased cardiac sympathetic activity assessed with ^123^I-*m*IBG. In addition, the cardiac sympathetic activity was associated with severity of heart failure (i.e., NT-proBNP and LVEF). In line with the lack of association between myocardial ^123^I-*m*IBG parameters and markers of inflammation, both were related to the prognosis of this specific CHF population.

Irrespective of the etiology of CHF, pro-inflammatory cytokines are implicated in the progression of CHF.^4^,^15^,^16^ In CHF patients, increased levels of TNF-α are associated with impaired LV function and consequently increased mortality.^17^,^18^ Although the exact mechanism of TNF-α in relation to CHF remains to be elucidated, it has been reported that TNF-α induces β-adrenergic receptor (βAR) desensitization.^19^ This phenomenon identifies a cross-talk between TNF-α and βAR function that at least in part contributes to a potential further reduction in cardiac contractility in CHF. In addition, increased levels of IL-6 have been reported in CHF patients and are correlated to plasma levels of NE (*R* = 0.839, *p* < 0.0001).^20^ In our study, plasma levels of IL-1β, IL-6, IL-10, IFN-α, IFN-β, and TNFR1 were below the detection limit. We consider it unlikely that our procedure failed to detect these cytokines as we took great care to process the blood samples quickly and limit activation. In addition, the earlier spike experiments for these cytokines yielded good recoveries and the internal standards were correct, A possible explanation for these undetectable levels could be the treatment with statins (hydroxymethylglutaryl-CoA reductase inhibitors), aspirin, ACE-Is, ARBs, mineralocorticoid receptor antagonists (MRAs), and beta-blockers. Statins have pleiotropic benefits independent of cholesterol levels including anti-inflammatory effects and it has been suggested that statins might reduce the production of TNF-α, IL-1, and IL-6.^21^^–^ 23 In addition, aspirin, ACE-Is/ARBs, MRAs, and beta-blockers have been shown to decrease plasma levels of cytokines.^24^ Consequently, the use of these drugs could have influenced the plasma levels of cytokines in our population. In addition, these findings may suggest that our stable CHF patients were optimally treated.

In line with others, we showed increased cardiac sympathetic activity (i.e., decreased late H/M ratio and increased ^123^I-*m*IBG WO) in a stable CHF population.^11^,^25^,^26^ However, in contrast to previous studies with IDCM,^27^,^28^ our study did not show a significant correlation between the most important markers of inflammation (i.e., TNF-α, IL-1β, and IL-6) and cardiac sympathetic activity. IL-1β and IL-6 levels were below the lower limit of quantification, whereas TNF-α was detectable, but did not show a correlation. In conclusion, in this population of stable, optimally treated CHF, markers of inflammation were subordinate to the more frequently used markers of prognosis in CHF (i.e., NT-proBNP, LVEF, NYHA class) in relation to sympathetic activity.

LVEF and NT-proBNP were moderately, but significantly, related to late H/M ratio. In addition, LVEF, NT-proBNP, and NYHA class were moderately related to ^123^I-*m*IBG WO. Recently, it has been shown that BNP modulates autonomic nervous function by inhibiting cardiac sympathetic activity in CHF.^29^ As in CHF, prolonged increased cardiac sympathetic activity has a detrimental effect on the contractility of the myocardium, this influences the LVEF. This is in line with the found negative association between LVEF and ^123^I-*m*IBG WO.

### Predictor of Cardiac Events

Increased cardiac sympathetic activity occurs early in the CHF disease process. Initially, βAR stimulation by the increased NE levels helps to compensate for impaired myocardial function, but long-term NE excess has detrimental effects on myocardial structure and gives rise to a downregulation of post-synaptic βARs.^30^ This downregulation leads to left ventricle remodeling and poor prognosis. In our study, decreased late H/M ratio was associated with CEs (Figure 2A). This is line with two large meta-analyses and a large prospective multicenter trial.^11^,^25^,^26^ However, in our study, not late H/M ratio, but CRP, MPO, IL12p40, and NT-proBNP were the only independent predictors of CEs.

CRP is regarded a non-specific marker for acute inflammation. Several studies have examined CRP levels in coronary heart disease and have demonstrated its prognostic value.^31^,^32^ Interestingly, in our study, CRP levels were significantly higher in patients with an event compared to whose without CEs (Figure 2B). Similar results were reported in IDCM patients.^33^ The past decade high-sensitive (hs)CRP has been introduced and is commonly used for cardiovascular risk stratification. Increased hsCRP is associated with increased risk for cardiovascular disease and mortality.^34^

MPO, an enzyme derived from neutrophilic granulocytes reflects inflammation and plaque destabilization. In addition to a possible candidate biomarker to predict future adverse events in patients with acute coronary syndromes (ASC) and myocardial infarction,^35^,^36^ MPO has been associated with the severity of CHF.^4^ In our study, the levels of MPO were elevated. Interestingly, activation of neutrophils leads to the release of preformed IL-8 and MPO. As we found no IL-8 in conjunction with MPO, we consider it unlikely that MPO was generated during processing of blood samples.^37^ In contrast to previous studies, 35,36 we found a negative association between these elevated levels of MPO and CEs, suggesting a protective mechanism of MPO. However, due to the relatively limited sample size, this extraordinary finding should be interpreted with great care.

IL-12p40 is elevated in an early stage of atherosclerosis both at the mRNA and protein level.^38^ Elevated levels of IL-12p40 have also been reported in patients with coronary artery disease (CAD).^39^,^40^ Interestingly, in a murine atherosclerosis study, aspirin reduced the levels of IL-12p40, suggesting the involvement of IL-12p40 on vascular inflammation.^41^ Our study showed a prognostic value of IL-12p40 in a stable CHF population. This association is most likely explained by the high percentage (i.e., 60%) of CAD in our CHF population.

NT-proBNP is a widely used powerful predictor of clinical outcome and a better marker for efficacy of drug treatment in patients with HF than other biomarkers or clinical parameters. Its clinical use has been endorsed in clinical practice guideline.^13^ NT-proBNP was an independent predictor of CEs, in line with previous results showing almost exponentially raising risk with increasing levels of NT-proBNP.^42^

Our study has several limitations. The major limitation of the study is the relative small number of patients included. This may have resulted in a limited statistical power. Therefore, the results of the study should be regarded as insightful but preliminary and larger studies are necessary to confirm our findings. Second, the study population was heterogeneous including ischemic and non-ischemic heart disease. Although markers of inflammation were elevated in both groups, it is conceivable that elevation of these markers is more pronounced in the presence of atherosclerosis (i.e., ischemia). Third, the event rate was relatively low in this stable heart failure population, resulting in non-significant result for the individual endpoints. Therefore, a combination of the individual endpoints into a single endpoint was chosen. Finally, treatment with statins, aspirin, ACE-I or ARBs, MRAs, and beta-blockers may have influenced cytokine production. However, treatment of CHF with these drugs is followed according to the international HF guidelines. Therefore, our results are an accurate reflection of this specific stable CHF population.

In conclusion, this study demonstrated that some markers of inflammation were undetectable most likely reflecting adequate medical treatment in clinically stable and optimally treated CHF patients. However, there is no association between detectable general markers of inflammation and cardiac sympathetic activity in this stable CHF population. This was corroborated by the fact that both markers of inflammation and cardiac sympathetic activity were prognostic indicators of CEs.

## New Knowledge Gained

The results of this study support the notion that inflammation and cardiac sympathetic activity are both important markers reflecting the multifactorial aspects of heart failure progression.

## Electronic supplementary material

Below is the link to the electronic supplementary material.

Supplementary material 1 (PPTX 300 kb)

## Abbreviations

CHF: Chronic heart failure
*m*IBG: Meta-iodobenzylguanidine
H/M ratio: Heart-to-mediastinum ratio
WO: Washout
LVEF: Left ventricle ejection fraction
NT-proBNP: N-terminal pro B-type Natriuretic Peptide
NYHA: New York Heart Association
CE: Cardiac event
CRP: C-reactive protein
MPO: Myeloperoxidase
TNF-α: Tumor necrosis factor-α
IL: Interleukin

## References

[1] Swedberg K, Viquerat C, Rouleau JL, Roizen M, Atherton B, Parmley WW (1984). Comparison of myocardial catecholamine balance in chronic congestive heart failure and in angina pectoris without failure. Am J Cardiol..

[2] Hasking GJ, Esler MD, Jennings GL, Burton D, Johns JA, Korner PI (1986). Norepinephrine spillover to plasma in patients with congestive heart failure: evidence of increased overall and cardiorenal sympathetic nervous activity. Circulation..

[3] Munger MA, Johnson B, Amber IJ, Callahan KS, Gilbert EM (1996). Circulating concentrations of proinflammatory cytokines in mild or moderate heart failure secondary to ischemic or idiopathic dilated cardiomyopathy. Am J Cardiol..

[4] Tang WH, Brennan ML, Philip K, Tong W, Mann S, Van Lente F (2006). Plasma myeloperoxidase levels in patients with chronic heart failure. Am J Cardiol..

[5] Verschure DO, Veltman CE, Manrique A, Somsen GA, Koutelou M, Katsikis A (2014). For what endpoint does myocardial 123I-MIBG scintigraphy have the greatest prognostic value in patients with chronic heart failure? Results of a pooled individual patient data meta-analysis. Eur Heart J Cardiovasc Imaging..

[6] Wieland DM, Brown LE, Les Rogers W, Worthington KC, Wu JL, Clinthorne NH (1981). Myocardial imaging with a radioiodinated norepinephrine storage analog. J Nucl Med..

[7] Agostini D, Carrio I, Verberne HJ (2009). How to use myocardial 123I-MIBG scintigraphy in chronic heart failure. Eur J Nucl Med Mol Imaging..

[8] Schofer J, Spielmann R, Schuchert A, Weber K, Schluter M (1988). Iodine-123 meta-iodobenzylguanidine scintigraphy: a noninvasive method to demonstrate myocardial adrenergic nervous system disintegrity in patients with idiopathic dilated cardiomyopathy. J Am Coll Cardiol..

[9] Wichter T, Hindricks G, Lerch H, Bartenstein P, Borggrefe M, Schober O (1994). Regional myocardial sympathetic dysinnervation in arrhythmogenic right ventricular cardiomyopathy. An analysis using 123I-meta-iodobenzylguanidine scintigraphy. Circulation..

[10] Shimizu M, Ino H, Yamaguchi M, Terai H, Hayashi K, Nakajima K (2002). Heterogeneity of cardiac sympathetic nerve activity and systolic dysfunction in patients with hypertrophic cardiomyopathy. J Nucl Med..

[11] Jacobson AF, Senior R, Cerqueira MD, Wong ND, Thomas GS, Lopez VA (2010). Myocardial iodine-123 meta-iodobenzylguanidine imaging and cardiac events in heart failure. Results of the prospective ADMIRE-HF (AdreView Myocardial Imaging for Risk Evaluation in Heart Failure) study. J Am Coll Cardiol..

[12] McGhie AI, Corbett JR, Akers MS, Kulkarni P, Sills MN, Kremers M (1991). Regional cardiac adrenergic function using I-123 meta-iodobenzylguanidine tomographic imaging after acute myocardial infarction. Am J Cardiol..

[13] Ponikowski P VA, Anker SD, Bueno H, Cleland JG, Coats AJ, Falk V, et al. (2016) ESC Guidelines for the diagnosis and treatment of acute and chronic heart failure: The Task Force for the diagnosis and treatment of acute and chronic heart failure of the European Society of Cardiology (ESC). Developed with the special contribution of the Heart Failure Association (HFA) of the ESC. Eur Heart Fail. 2016; epub ahead of print. 10.1002/ejhf.592 27207191

[14] Flotats A, Carrió I, Agostini D, Le Guludec D, Marcassa C, Schaffers M (2010). Proposal for standardization of 123I-metaiodobenzylguanidine (MIBG) cardiac sympathetic imaging by the EANM Cardiovascular Committee and the European Council of Nuclear Cardiology. Eur J Nucl Med Mol Imaging..

[15] Stumpf C, Lehner C, Yilmaz A, Daniel WG, Garlichs CD (2003). Decrease of serum levels of the anti-inflammatory cytokine interleukin-10 in patients with advanced chronic heart failure. Clin Sci..

[16] Anker SD, von Haehling S (2004). Inflammatory mediators in chronic heart failure: an overview. Heart..

[17] Rodriguez-Reyna TS, Arrieta O, Castillo-Martinez L, Orea-Tejeda A, Guevara P, Rebollar V (2005). Tumour Necrosis Factor alpha and Troponin T as predictors of poor prognosis in patients with stable heart failure. Clin Invest Med..

[18] Dunlay SM, Weston SA, Redfield MM, Killian JM, Roger VL (2008). Tumor necrosis factor-α and mortality in heart failure: a community study. Circulation..

[19] Vasudevan NT, Mohan ML, Gupta MK, Martelli EE, Hussain AK, Qin Y (2013). Gbetagamma-independent recruitment of G-protein coupled receptor kinase 2 drives tumor necrosis factor alpha-induced cardiac beta-adrenergic receptor dysfunction. Circulation..

[20] Tsutamoto T, Hisanaga T, Wada A, Maeda K, Ohnishi M, Fukai D (1998). Interleukin-6 spillover in the peripheral circulation increases with the severity of heart failure, and the high plasma level of interleukin-6 is an important prognostic predictor in patients with congestive heart failure. J Am Coll Cardiol..

[21] Lyngdoh T, Vollenweider P, Waeber G, Marques-Vidal P (2011). Association of statins with inflammatory cytokines: a population-based Colaus study. Atherosclerosis..

[22] Sygitowicz G, Maciejak A, Piniewska-Juraszek J, Pawlak M, Gora M, Burzynska B (2016). Interindividual variability of atorvastatin treatment influence on the MPO gene expression in patients after acute myocardial infarction. Acta Biochim Pol..

[23] Rauchhaus M, Coats AJS, Anker SD (2000). The endotoxin-lipoprotein hypothesis. Lancet..

[24] Esposito CT, Varahan S, Jeyaraj D, Lu Y, Stambler BS (2013). Spironolactone improves the arrhythmogenic substrate in heart failure by preventing ventricular electrical activation delays associated with myocardial interstitial fibrosis and inflammation. J Cardiovasc Electrophysiol..

[25] Verberne HJ, Brewster LM, Somsen GA, van Eck-Smit BL (2008). Prognostic value of myocardial 123I-metaiodobenzylguanidine (MIBG) parameters in patients with heart failure: a systematic review. Eur Heart J..

[26] Nakata T, Nakajima K, Yamashina S, Yamada T, Momose M, Kasama S (2013). A pooled analysis of multicenter cohort studies of 123I-mIBG imaging of sympathetic innervation for assessment of long-term prognosis in heart failure. JACC Cardiovasc Imaging..

[27] Parthenakis FI, Patrianakos A, Prassopoulos V, Papadimitriou E, Nikitovic D, Karkavitsas NS (2003). Relation of cardiac sympathetic innervation to proinflammatory cytokine levels in patients with heart failure secondary to idiopathic dilated cardiomyopathy. Am J Cardiol..

[28] Messias LR, Carreira MA, Miranda SM, Azevedo JC, Benayon PC, Rodrigues RC (2013). Do interleukin-1beta levels correlate with MIBG and exercise parameters in heart failure?. Arq Bras Cardiol..

[29] Brunner-La Rocca HP, Kaye DM, Woods RL, Hastings J, Esler MD (2001). Effects of intravenous brain natriuretic peptide on regional sympathetic activity in patients with chronic heart failure as compared with healthy control subjects. J Am Coll Cardiol..

[30] Mardon K, Montagne O, Elbaz N, Malek Z, Syrota A, Dubois-Rande JL (2003). Uptake-1 carrier downregulates in parallel with the beta-adrenergic receptor desensitization in rat hearts chronically exposed to high levels of circulating norepinephrine: implications for cardiac neuroimaging in human cardiomyopathies. J Nucl Med..

[31] Anzai T, Yoshikawa T, Shiraki H, Asakura Y, Akaishi M, Mitamura H (1997). C-reactive protein as a predictor of infarct expansion and cardiac rupture after a first Q-wave acute myocardial infarction. Circulation..

[32] Pietila K, Harmoinen A, Teppo AM (1991). Acute phase reaction, infarct size and in-hospital morbidity in myocardial infarction patients treated with streptokinase or recombinant tissue type plasminogen activator. Ann Med..

[33] Kaneko K, Kanda T, Yamauchi Y, Hasegawa A, Iwasaki T, Arai M (1999). C-reactive protein in dilated cardiomyopathy. Cardiology..

[34] Parrinello CM, Lutsey PL, Ballantyne CM, Folsom AR, Pankow JS, Selvin E (2015). Six-year change in high-sensitivity C-reactive protein and risk of diabetes, cardiovascular disease, and mortality. Am Heart J..

[35] Stankovic S, Asanin M, Trifunovic D, Majkic-Singh N, Ignjatovic S, Mrdovic I (2012). Time-dependent changes of myeloperoxidase in relation to in-hospital mortality in patients with the first anterior ST-segment elevation myocardial infarction treated by primary percutaneous coronary intervention. Clin Biochem..

[36] Brennan M-L, Penn MS, Van Lente F, Nambi V, Shishehbor MH, Aviles RJ (2003). Prognostic value of myeloperoxidase in patients with chest pain. N Engl J Med..

[37] Altstaedt J, Kirchner H, Rink L (1996). Cytokine production of neutrophils is limited to interleukin-8. Immunology..

[38] Lee T-S, Yen H-C, Pan C-C, Chau L-Y (1999). The role of interleukin 12 in the development of atherosclerosis in ApoE-deficient mice. Arterioscler Thromb Vasc Biol..

[39] Fernandes JL, Mamoni RL, Orford JL, Garcia C, Selwyn AP, Coelho OR (2004). Increased Th1 activity in patients with coronary artery disease. Cytokine..

[40] Yamashita H, Shimada K, Seki E, Mokuno H, Daida H (2003). Concentrations of interleukins, interferon, and C-reactive protein in stable and unstable angina pectoris. Am J Cardiol..

[41] Cyrus T, Sung S, Zhao L, Funk CD, Tang S, Praticò D (2002). Effect of low-dose aspirin on vascular inflammation, plaque stability, and atherogenesis in low-density lipoprotein receptor-deficient mice. Circulation..

[42] Cleland JG, McMurray JJ, Kjekshus J, Cornel JH, Dunselman P, Fonseca C (2009). Plasma concentration of amino-terminal pro-brain natriuretic peptide in chronic heart failure: prediction of cardiovascular events and interaction with the effects of rosuvastatin: a report from CORONA (Controlled Rosuvastatin Multinational Trial in Heart Failure). J Am Coll Cardiol..

