# Supplementary material for: Myocardial 123I-mIBG scintigraphy in relation to markers of inflammation and long-term clinical outcome in patients with stable chronic heart failure
Source: J Nucl Cardiol. 2016 Nov 17;25(3):845–53. doi: 10.1007/s12350-016-0697-7 (PMC5966475; doi:10.1007/s12350-016-0697-7)
Supplement: Supplementary file 1 — Supplementary material 1 (PPTX 300 kb) [file 12350_2016_697_MOESM1_ESM.pptx]

## Slide 1
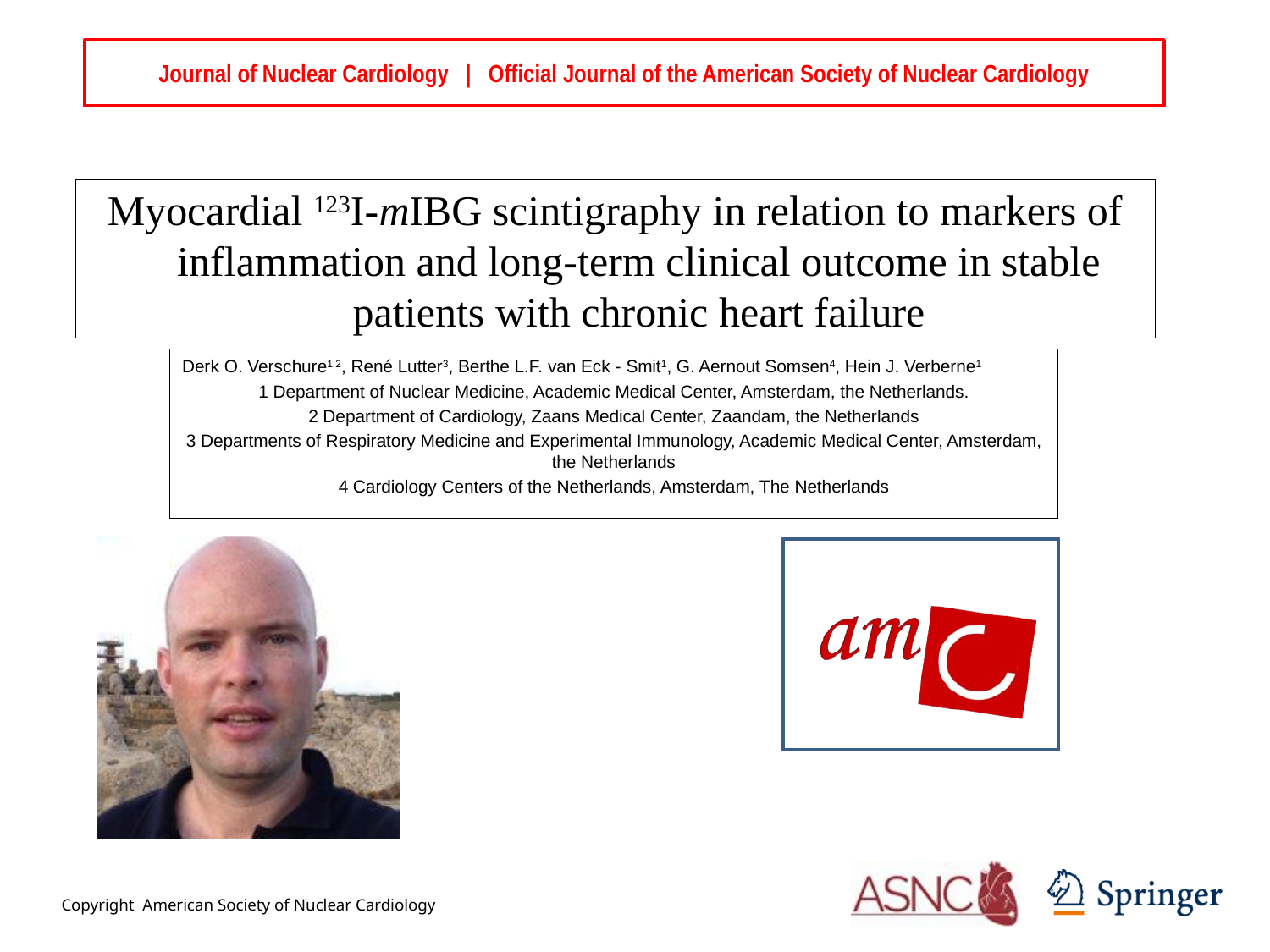

Journal of Nuclear Cardiology | Official Journal of the American Society of Nuclear Cardiology
# Myocardial 123I-mIBG scintigraphy in relation to markers of inflammation and long-term clinical outcome in stable patients with chronic heart failure
Derk O. Verschure1,2, René Lutter3, Berthe L.F. van Eck - Smit1, G. Aernout Somsen4, Hein J. Verberne1
1 Department of Nuclear Medicine, Academic Medical Center, Amsterdam, the Netherlands.
2 Department of Cardiology, Zaans Medical Center, Zaandam, the Netherlands
3 Departments of Respiratory Medicine and Experimental Immunology, Academic Medical Center, Amsterdam, the Netherlands
4 Cardiology Centers of the Netherlands, Amsterdam, The Netherlands
Copyright American Society of Nuclear Cardiology

## Slide 2
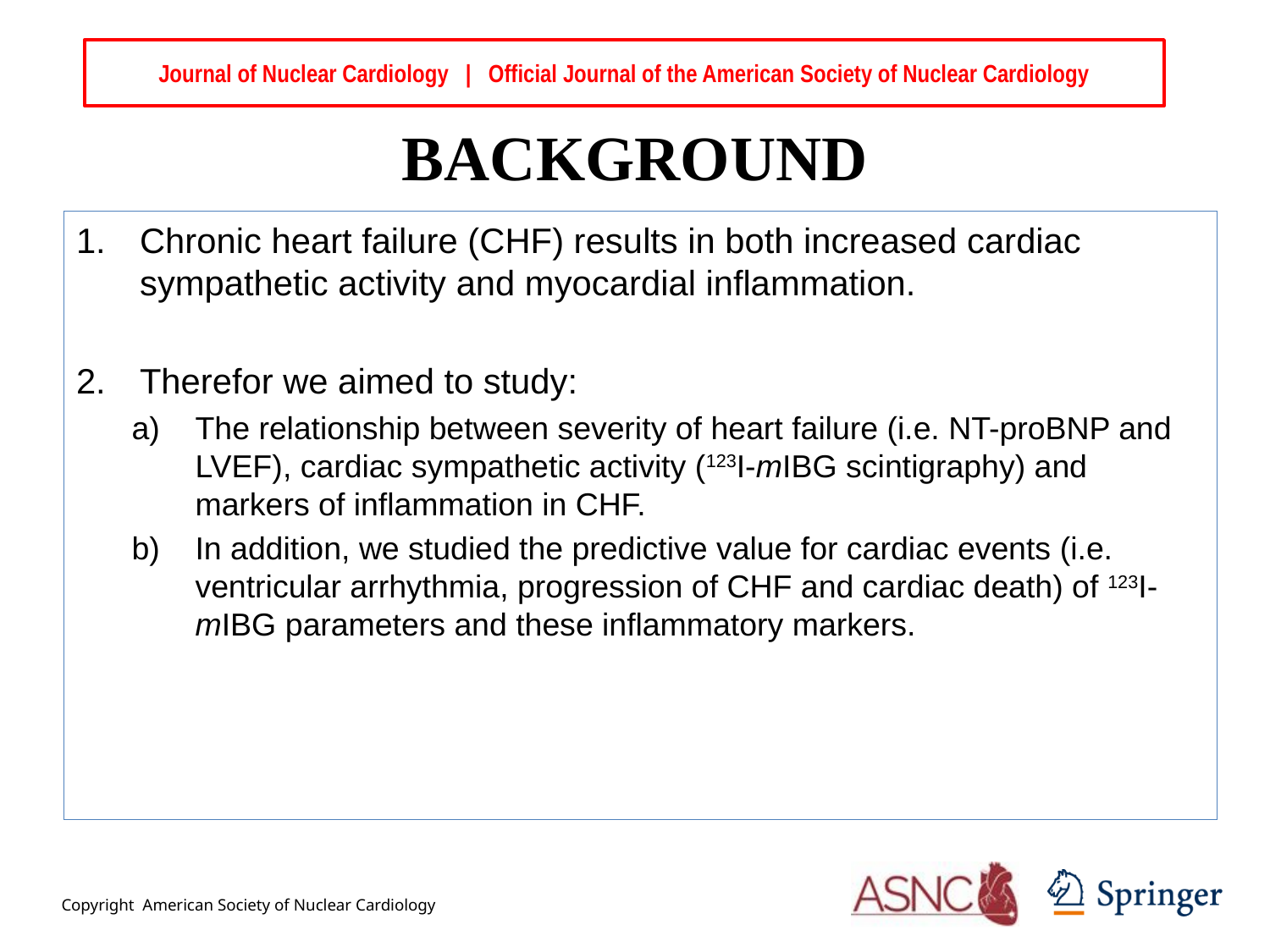

Journal of Nuclear Cardiology | Official Journal of the American Society of Nuclear Cardiology
# BACKGROUND
Chronic heart failure (CHF) results in both increased cardiac sympathetic activity and myocardial inflammation.
Therefor we aimed to study:
The relationship between severity of heart failure (i.e. NT-proBNP and LVEF), cardiac sympathetic activity (123I-mIBG scintigraphy) and markers of inflammation in CHF.
In addition, we studied the predictive value for cardiac events (i.e. ventricular arrhythmia, progression of CHF and cardiac death) of 123I-mIBG parameters and these inflammatory markers.
Copyright American Society of Nuclear Cardiology

## Slide 3
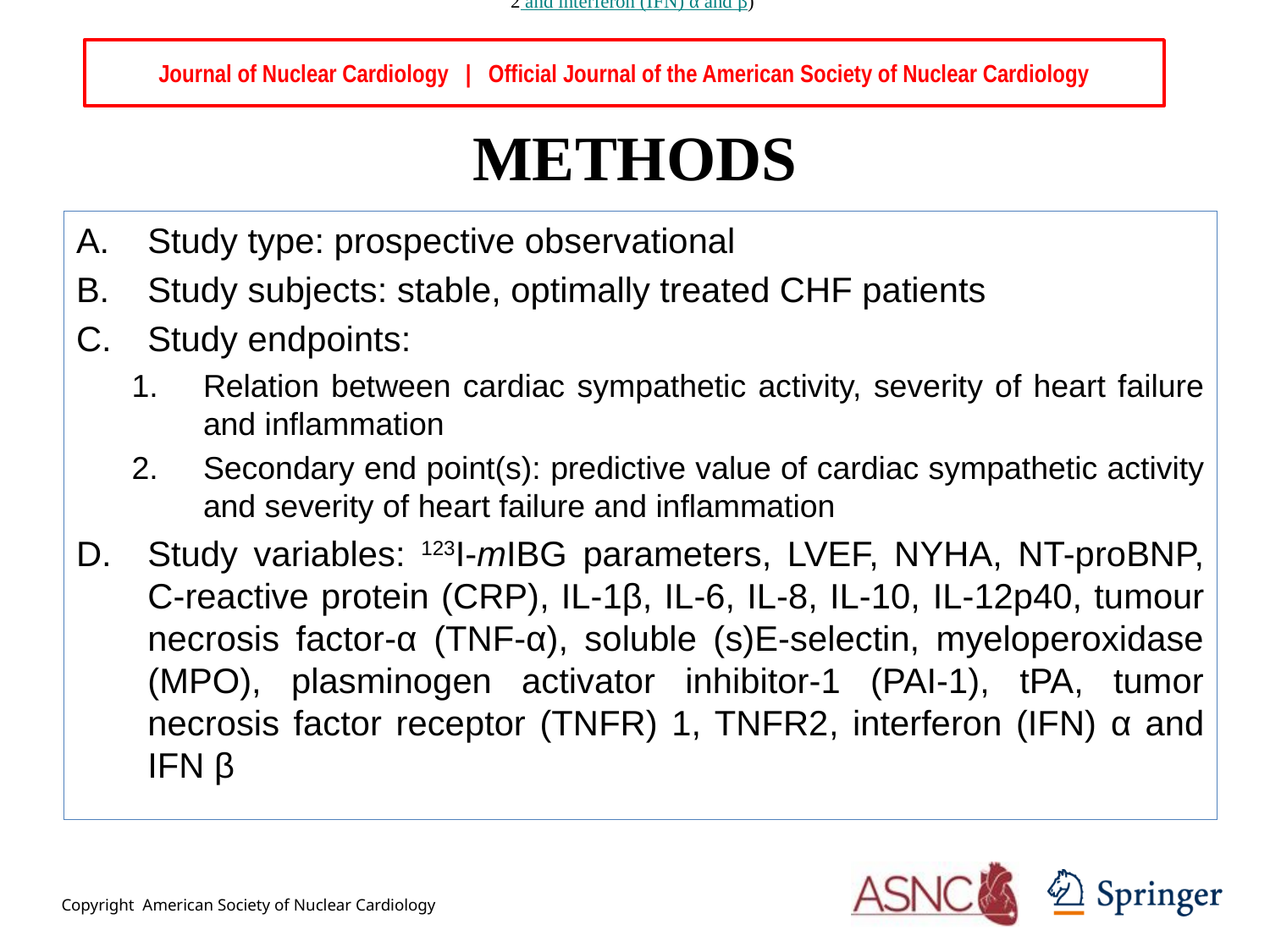

2 and interferon (IFN) α and β)
Journal of Nuclear Cardiology | Official Journal of the American Society of Nuclear Cardiology
# METHODS
Study type: prospective observational
Study subjects: stable, optimally treated CHF patients
Study endpoints:
Relation between cardiac sympathetic activity, severity of heart failure and inflammation
Secondary end point(s): predictive value of cardiac sympathetic activity and severity of heart failure and inflammation
Study variables: 123I-mIBG parameters, LVEF, NYHA, NT-proBNP, C-reactive protein (CRP), IL-1β, IL-6, IL-8, IL-10, IL-12p40, tumour necrosis factor-α (TNF-α), soluble (s)E-selectin, myeloperoxidase (MPO), plasminogen activator inhibitor-1 (PAI-1), tPA, tumor necrosis factor receptor (TNFR) 1, TNFR2, interferon (IFN) α and IFN β
Copyright American Society of Nuclear Cardiology

## Slide 4
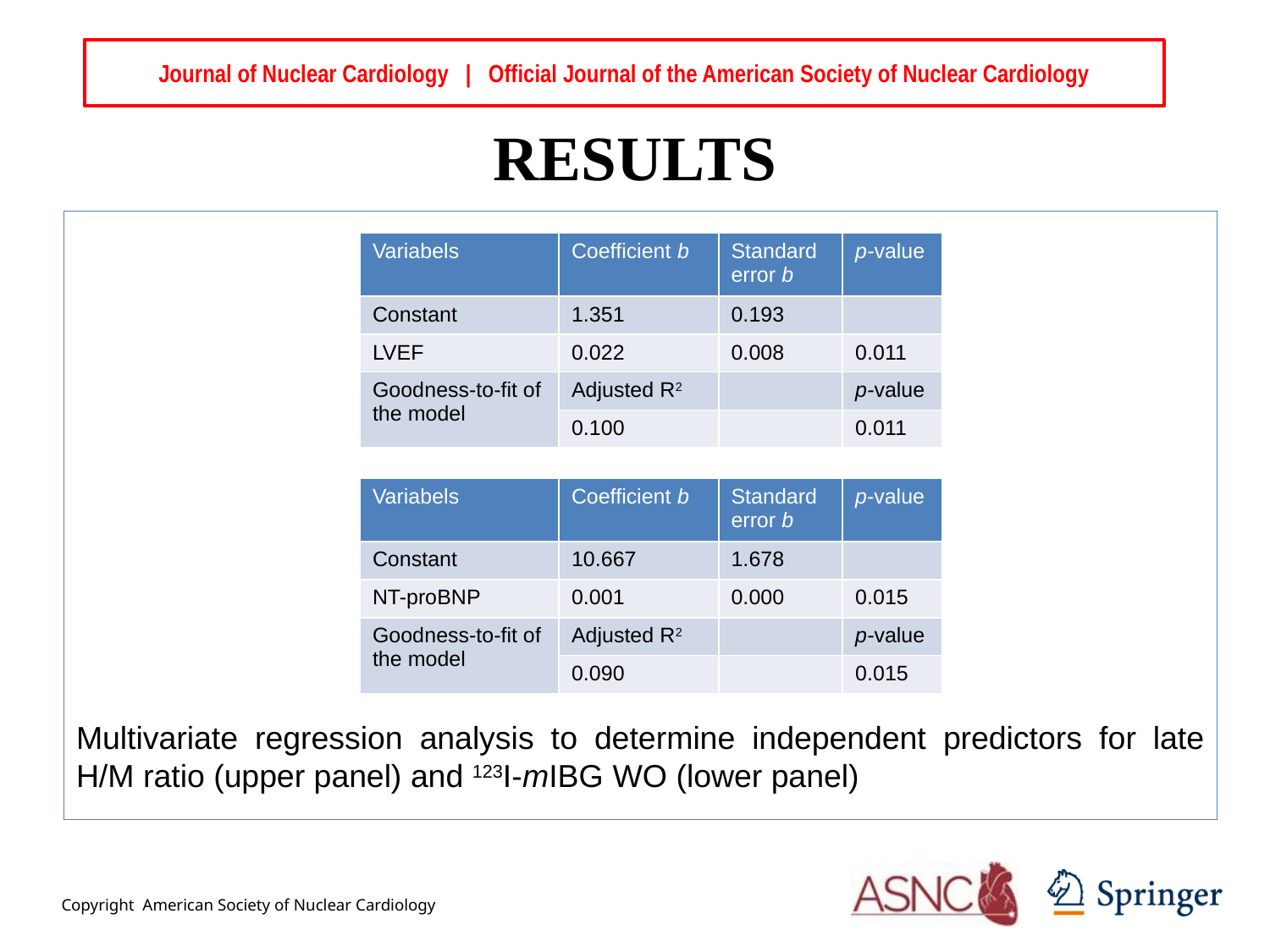

Journal of Nuclear Cardiology | Official Journal of the American Society of Nuclear Cardiology
# RESULTS
Multivariate regression analysis to determine independent predictors for late H/M ratio (upper panel) and 123I-mIBG WO (lower panel)
| Variabels | Coefficient b | Standard error b | p-value |
| --- | --- | --- | --- |
| Constant | 1.351 | 0.193 | |
| LVEF | 0.022 | 0.008 | 0.011 |
| Goodness-to-fit of the model | Adjusted R2 | | p-value |
| | 0.100 | | 0.011 |
| Variabels | Coefficient b | Standard error b | p-value |
| --- | --- | --- | --- |
| Constant | 10.667 | 1.678 | |
| NT-proBNP | 0.001 | 0.000 | 0.015 |
| Goodness-to-fit of the model | Adjusted R2 | | p-value |
| | 0.090 | | 0.015 |
Copyright American Society of Nuclear Cardiology

## Slide 5
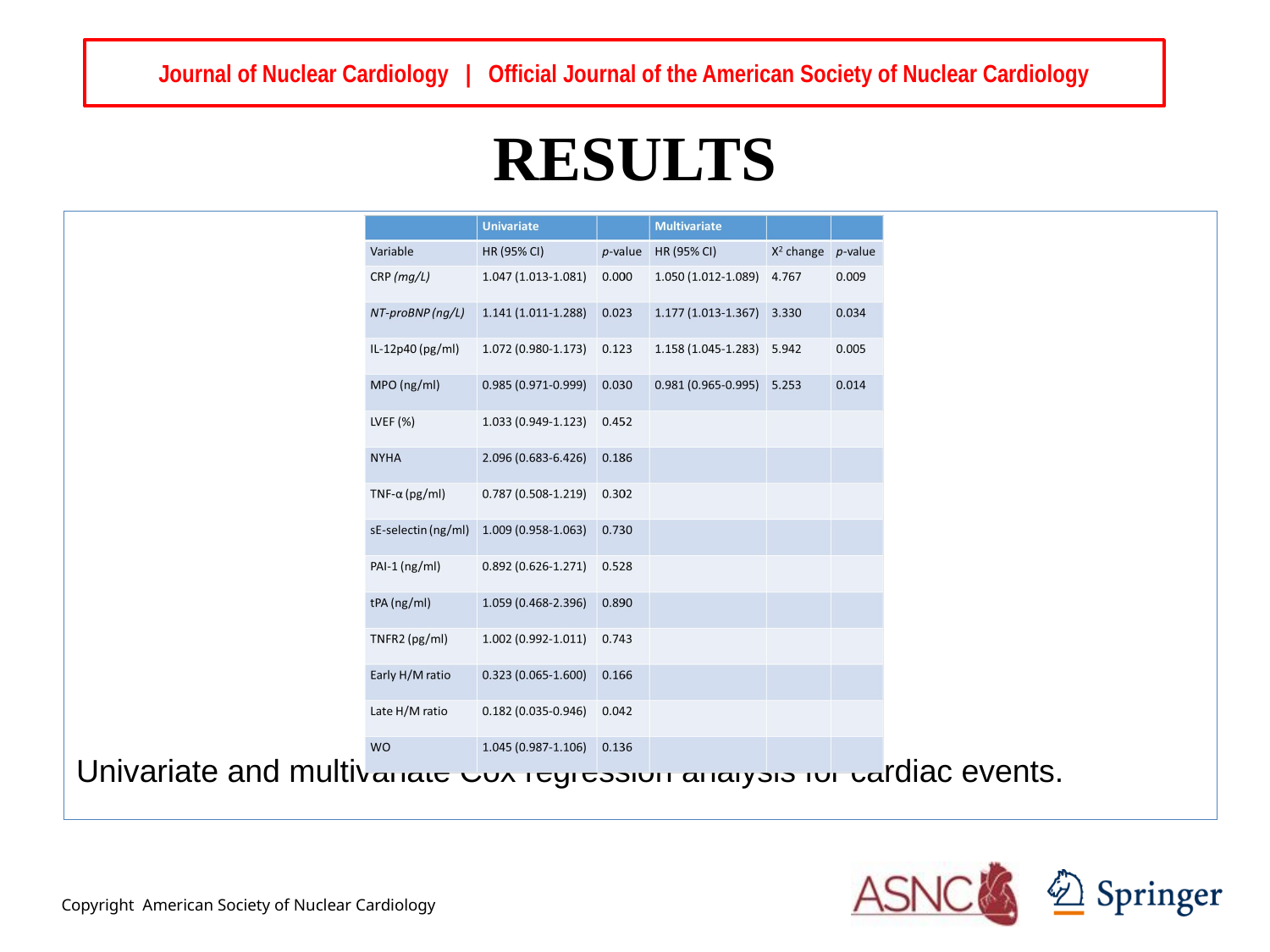

Journal of Nuclear Cardiology | Official Journal of the American Society of Nuclear Cardiology
# RESULTS
Univariate and multivariate Cox regression analysis for cardiac events.
Copyright American Society of Nuclear Cardiology

## Slide 6
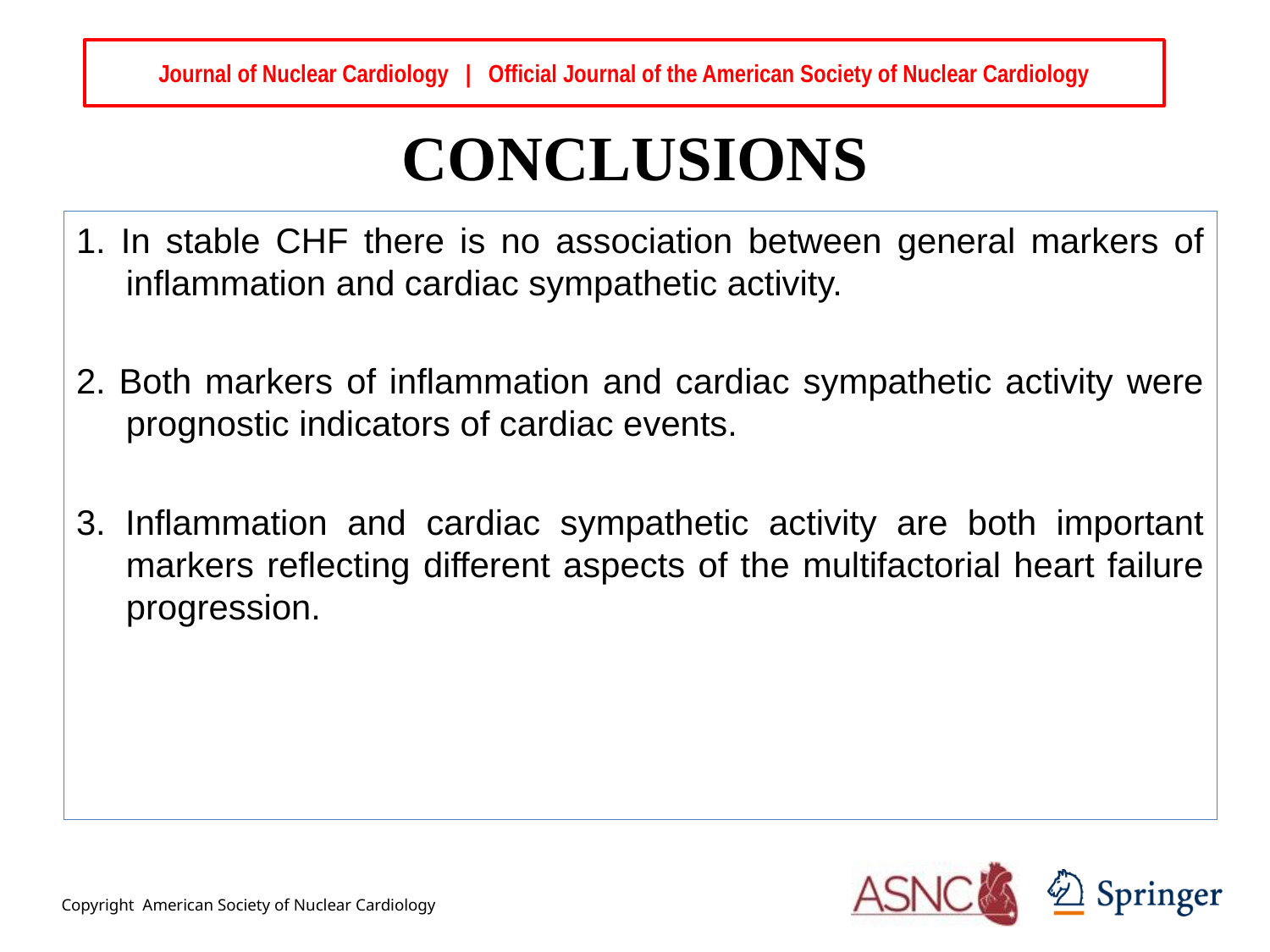

Journal of Nuclear Cardiology | Official Journal of the American Society of Nuclear Cardiology
# CONCLUSIONS
1. In stable CHF there is no association between general markers of inflammation and cardiac sympathetic activity.
2. Both markers of inflammation and cardiac sympathetic activity were prognostic indicators of cardiac events.
3. Inflammation and cardiac sympathetic activity are both important markers reflecting different aspects of the multifactorial heart failure progression.
Copyright American Society of Nuclear Cardiology
